# Novelties in Macrofungi of the Tropical Montane Cloud Forest in Mexico

**DOI:** 10.3390/jof9040477

**Published:** 2023-04-15

**Authors:** Ricardo Valenzuela, Isolda Luna-Vega, Michelle Martínez-Pineda, César Ramiro Martínez-González, Jesús García-Jiménez, Javier de la Fuente, Silvia Bautista-Hernández, Salvador Acosta-Castellanos, Tania Raymundo

**Affiliations:** 1 Laboratorio de Micología, Departamento de Botánica, Instituto Politécnico Nacional, Escuela Nacional de Ciencias Biológicas, Mexico City 11340, CDMX, Mexico; 2 Laboratorio de Biogeografía y Sistemática, Departamento de Biología Evolutiva, Facultad de Ciencias, Universidad Nacional Autónoma de México, Mexico City 04510, CDMX, Mexico; 3 Instituto de Horticultura, Departamento de Fitotecnia, Universidad Autónoma Chapingo, Km 38.5 Carretera Federal México-Texcoco, Texcoco 56230, Estado de México, Mexico; 4 Tecnológico Nacional de México, Instituto Tecnológico de Ciudad Victoria, Blvd. Emilio Portes Gil #1301 Pte., Ciudad Victoria 87010, Tamaulipas, Mexico; 5 Colegio de Posgraduados, Km 36.5, Montecillo, Texcoco 56230, Estado de México, Mexico

**Keywords:** Agaricomycetes, new species, phylogeny, taxonomy, *Bondarzewia*, *Gymnopilus*, *Serpula*, *Sparassis*

## Abstract

The tropical montane cloud forest in Mexico is the most diverse and threatened ecosystem. Mexican macrofungi numbers more than 1408 species. This study described four new species of Agaricomycetes (*Bondarzewia*, *Gymnopilus*, *Serpula*, *Sparassis*) based on molecular and morphological characteristics. Our results support that Mexico is among the most biodiverse countries in terms of macrofungi in the Neotropics.

## 1. Introduction

In Mexico, the tropical montane cloud forest (TMCF), also known as a cloud forest or “bosque mesófilo de montaña”, groups together a set of physiognomically heterogeneous plant communities. The canopy of these forests is usually composed of evergreen trees, but the medium and low-strata trees are deciduous. The high abundance of epiphytes and ferns gives the forest an exuberant appearance [1,2]. The TMCF features a mixture of Holarctic affinities in the upper arboreal stratum and meridional affinities in the medium, low, shrub, and herbaceous strata [3,4,5]. These forests inhabit mountainous areas, mainly between 600 (1200) and 2500 (3200) m above sea level, with seasonal persistence of high relative humidity and rainfall that ranges from 2000 to 4000 (5300) mm per year. The soils are mainly acidic and prosper in temperate climates [6,7,8]. One of the most outstanding characteristics of these forests is that the arboreal canopy intercepts and condenses the fog, which precipitates and contributes about 50% of the total local precipitation [9].

Mexican TMCF presents a great β diversity, showing a high turnover of species [10]. In Mexico, this ecosystem has the highest number of species per unit area (in Oaxaca, up to 75 spp/0.1 ha: div. α). Mexico has 2500 almost exclusive vascular plant species in less than 1% of the territory [1,11]. The forest contains approximately 10% of the total flora of Mexico, of which 30% of the species are endemic [1].

**Figure 1:**
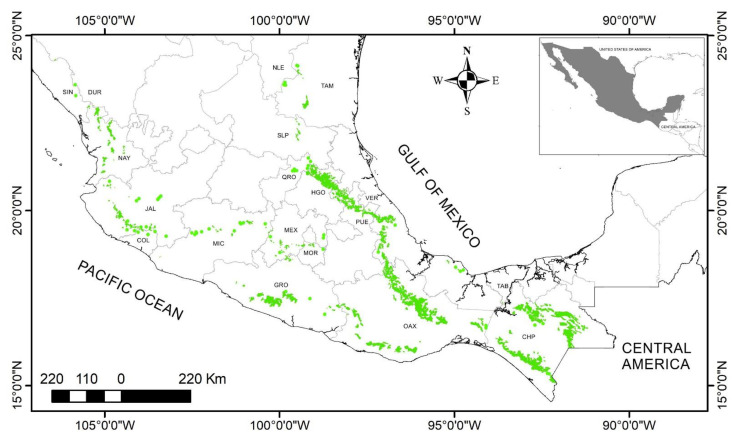
Map of Mexico showing the geographic location of the TMCF (modified from [11]). Mexican state abbreviations are as follows: CHP, Chiapas; COL, Colima; DUR, Durango; GRO, Guerrero; HGO, Hidalgo; JAL, Jalisco; MEX, México; MIC, Michoacán; MOR, Morelos; NAY, Nayarit; NLE, Nuevo León; OAX, Oaxaca; PUE, Puebla; QRO, Querétaro; SIN, Sinaloa; SLP, San Luis Potosí; TAB, Tabasco; TAM, Tamaulipas; and VER, Veracruz.

TMCF is one of the most threatened ecosystems in the country [3,12,13] (Figure 1). The demographic explosion, clandestine logging, coffee cultivation, cattle grazing, and semi-nomadic agriculture have caused a drastic decrease in its extension in recent decades. The area occupied by this forest was reduced to less than a tenth in twenty years [13]. The extensive use of these forests began after the conquest, with the displacement of indigenous people to more abrupt lands, and during the Porfiriato (1877–1911) with large-scale coffee plantations [14]. In addition, the TMCF occupies fragile and acidic low-fertility soils [5,15]. 

In the last 70 years in Mexico, various studies have been carried out on the TMCF [1,5,6,16,17,18,19,20,21,22,23,24,25,26,27]. The floristic associations in the TMCF are unique and maintain a variable degree of relationships. This variation in the TMCF of Mexico increases when it is considered on the regional scale [28]. The Mexican states with the highest forest coverage are Oaxaca, Chiapas, and Hidalgo [13].

The diversity of fungi is estimated at 1.5–3.8 million species worldwide, of which 120,000 have been described [29]; macrofungi represent 18% of this diversity. Fungi have not been studied thoroughly, and it has been estimated that there could be as many as 53,000 to 110,000 more species [30]. This number may vary if we consider cryptic and species complexes that have been clarified from polyphasic or comprehensive studies [31].

The study of macrofungi in Mexico is still incipient. The groups best studied are Pezizales and Xylariales for Ascomycota, while Agaricales, Boletales, Dacrymycetales, Hymenochaetales, Polyporales, and Russulales are the most recorded in Basidiomycota. Monographic studies have only been undertaken for 60 genera [32]. In the Comisión Nacional para el Estudio y Conocimiento de la Biodiversidad (CONABIO) catalog [33], Cifuentes (2008) [34] registered 2135 fungi species from Mexico. 

Recently, Del Olmo et al. (2017) [35] analyzed 6349 records of fungi ascribed to 2962 species from the tropical montane cloud forests (Mexico, Guatemala, Belize, Brazil, Colombia, Costa Rica, Panama, Venezuela), of which 220 taxa were described initially from this ecosystem. These authors indicated that Mexico presents 36% of these records (1274 species).

The genera *Bondarzewia*, *Gymnopilus*, *Serpula*, and *Sparassis* belong to the class Agaricomycetes in the orders Russulales, Agaricales, Boletales, and Polyporales, respectively. The order Russulales is the most diverse morphologically. It contains a remarkable variety of sporophore forms, including resupinate, discoid, effused-reflexed, clavarioid, pileate, or gasteroid and hymenophore configurations go from smooth, poroid, hydnoid, and lamellate to labyrinthoid forms [36]. This order contains ten families, 98 genera, and 4436 species. The family Bondarzewiaceae and genus *Bondarzewia* were recognized phylogenetically with well-supported posterior probability (PP) values [37]. Among the Russulales, *Bondarzewia* is characterized morphologically by strongly amyloid and ornamented basidiospores [38]. 

The Agaricales or euagarics is the largest clade of Agaricomycetes, including more than half of all known species. Most are agaricoid fungi, including also clavarioid and gasteroid fungi [38]. The order contains 38 families, 508 genera, and 17,291 species. The family Hymenogastraceae and the genus *Gymnopilus* have been recognized phylogenetically based on well-supported PP values [37]. The family Hymenogastraceae includes nine agaricoid genera and one gasteroid genus with ornamented or smooth, brown spores. There are no morphological synapomorphies that distinguish the members of this group. 

The Boletales includes conspicuous stipitate-pileate forms with tubular or lamellate hymenophores but also includes gasteroid, resupinate, or crust-like fungi that produce smooth, merulioid, hydnoid, or polyporoid hymenophores [39]. The order contains 16 families, 141 genera, and 2022 species. The family Serpulaceae and the genus *Serpula* have been recognized phylogenetically with well-supported PP values [37]. 

The saprotrophs species (or wood decay fungi) of Boletales developed a unique mode of brown rot called “Coniophoraceae-rot”. Serpulaceae comprises three genera: two of them are ectomycorrhizal fungi and *Serpula* produces brown rot [39]. Given the diversity of fruiting body forms, we assume that there has been extensive homoplasy in the evolution of Boletales. However, no apparent morphological character distinguishes the group, which is only diagnosed by molecular sequences. 

The Polyporales include various basidiocarp types and hymenophore configurations, including bracket-shaped, effused resupinate, stipitate with poroid, lamellate, or smooth hymenophores. Few species produce shelf-like or flabellate clusters of overlapping basidiocarps [40]. The order contains 18 families, 285 genera, and 2544 species. The family Sparassidaceae and the genus *Sparassis* have been recognized phylogenetically with well-supported PP values; analyzed the combination of *rpb1* and ribosomal RNA genes [37,41]; these authors discovered a robust resolution of many clades, including 18 families. However, these authors mentioned that some nodes remain weakly supported; perhaps because numerous taxa have not been sampled yet. The researchers [41] found that macroscopic and microscopic characters are variable and are present in several families of Polyporales [41]. Variations and transitions among basidiocarp types exist, and no morphological synapomorphy unites the Polyporales [40]. The most common ‘‘polyporoid’’ basidiocarp type also has convergently evolved in at least 11 additional orders of Agaricomycetes [39].

In Mexico, particular emphasis has been placed on studying the macrofungi of this type of forest. Many have been classified as in danger of extinction due to the high anthropogenic action [1,4]. The main objective of this study is (1) to describe new species and (2) to recognize and publicize the Mexican TMCF fungal richness. The present contribution aims to describe species distributed in the Mexican TMCF, an ecosystem that is in danger of extinction.

## 2. Material and Methods

### 2.1. Morphological Studies

The specimens were deposited in the “Dr. Gastón Guzmán Huerta” fungi collection of the Herbarium of the Escuela Nacional de Ciencias Biológicas, Instituto Politécnico Nacional, Mexico City, Mexico (ENCB) and “Jose Castillo Tovar” of the Instituto Tecnológico de Ciudad Victoria (ITCV). Georeferences were obtained with Garmin e-Trex 32X GPS (Garmin Ltd., Olathe, USA). Color codes follow Kornerup and Wanscher [42] and Bessette et al. [43]. Microscopic observations were taken from tissues rehydrated in 5% aqueous KOH and Melzer’s reagent; basidiospore dimensions include the ornamentation. Macroscopic features were photographed with a Nikon D7000 camera, (Nikon Corporation, Tokyo, Japan) and the micrographs were with a Sony DSCWX350 camera (Tokyo, Japan). Additionally, scanning electron microscopy (SEM; Hitachi SU1510, Hitachi, Japan) was used to observe the detail of the spore wall. The meanings of taxonomic terms are based on [44].

### 2.2. Extraction, Amplification, and Sequencing

We obtained the DNA from herbaria material. The CTAB protocol of [45] was used to extract genomic DNA. The DNA was quantified with a Nanodrop 2000c (Thermo Scientific^TM^, Wilmington, CA, USA). Then, we prepared dilutions from each sample at 20 ng/µL to amplify the following regions: the internal transcribed spacer rDNA-ITS1 5.8S rDNA-ITS2 (ITS), the larger nuclear subunit ribosomal DNA (*nLSU*), the second largest subunit of the RNA polymerase II gene (*rpb2*), the region of the small mitochondrial subunit (SSU), the subunit (*atp6*)*,* and the translation elongation factor 1-α (*tef1*) (Table 1). The sequences used for each species are described in the corresponding section. The reaction mixture for PCRs was performed on a final volume of 15 µL containing 1x buffer, 0.8 mM dNTPs mix, 20 pmol of each primer, 2 units of GoTaq DNA (Promega Corporation, Madison, WI, USA), and 100 ng of template DNA. The PCR products were verified by agarose gel electrophoresis. The gels were run for 1 h at 95 V cm^−3^ in 1.5% agarose and 1× TAE buffer (Tris Acetate-EDTA). The gel was stained with GelRed (Biotium, Fremont, CA, USA), and the bands were visualized in an Infinity 3000 transilluminator (Vilber Lourmat, Eberhardzell, Germany). The amplified products were purified with the ExoSAP Purification kit (Affymetrix Inc., Santa Clara, CA, USA), following the manufacturer’s instructions. Then, they were quantified and prepared for the sequence reaction using a BigDye Terminator v.3.1 (Applied Biosystems, Foster City, CA, USA). These products were sequenced in both directions with an Applied Biosystem model 3730XL (Applied BioSystems, Foster City, CA, USA) at the Instituto de Biología of the Universidad Nacional Autónoma de México (UNAM). The sequences obtained were compared with the original chromatograms to detect and correct possible reading errors. The sequences of both strands of each of the genes were analyzed, edited, and assembled using BioEdit v. 7.0.5 [46] to generate a consensus sequence and then compared with those deposited in GenBank (2020) using the tool BLASTN v. 2.2.9 [47,48,49,50,51].

### 2.3. Phylogenetic Analysis

The alignment obtained to explore the phylogenetic relationships of the new species of *Gymnopilus* was based on the taxonomic sampling employed by [52,53] (Table 2). The ITS region was aligned using the online version of MAFFT v. 7 [54,55,56]. Alignments were reviewed in PhyDE v.10.0 [57], followed by minor manual adjustments to ensure character homology between taxa. The matrix was composed of 88 taxa (697 characters). 

In the case of the new species of *Serpula,* we followed the taxonomic sampling of [58] (Table 3). First, the ITS region was aligned using the online version of MAFFT v. 7 [54,55,56]. Next, alignment was reviewed in PhyDE v.10.0 [57], followed by minor manual adjustments to ensure character homology between taxa. The matrix was composed of 46 taxa (700 characters). 

To analyze the new species of *Sparassis,* we followed the taxonomic sampling of [59] (Table 4). Each gene region was independently aligned using the online version of MAFFT v. 7 [54,55,56]. Alignment was reviewed in PhyDE v.10.0 [57], followed by minor manual adjustments to ensure character homology between taxa. The matrix was formed for ITS by 20 taxa (690 characters) and LSU by 19 taxa (831 characters). We established two partitioning schemes, one for the ITS and one for the LSU, using the option to minimize the stop codon with Mesquite v 3.70 [60].

In the case of the new species of *Bondarzewia,* an alignment was made based on the taxonomic sampling employed by [61] (Table 5). Each gene region was independently aligned using the online version of MAFFT v. 7 [54,55,56]. The alignment was reviewed in PhyDE v.10.0 [57], followed by minor manual adjustments to ensure the character homology among taxa. The matrix was formed for ITS by 30 taxa (690 characters), for LSU by 26 taxa (831 characters), and for mtSSU by 21 taxa (640 characters), while translation elongation factor 1-α (*tef1*) was formed by 23 taxa (670 characters). Finally, the aligned matrices were concatenated into a single matrix (30 taxa, 2831 characters). Six partitioning schemes were established: one for the ITS, one for the nLSU, one SSU, and three for the *tef1* gene region, using the option to minimize the stop codon with Mesquite v3.70 [60].

Phylogenetic inferences were estimated with maximum likelihood in RAxML v. 8.2.10 [62] with a GTR + G model of nucleotide substitution. In addition, 10,000 nonparametric rapid bootstrap pseudoreplicates assessed the branch support that was run with the GTRGAMMA model. For Bayesian posterior probability, the best evolutionary model for alignment was sought using the Partition Finder [63,64,65]. Phylogeny analyses were performed using MrBayes v. 3.2.6 × 64 [66]. The information block for the matrix includes two simultaneous runs, four Montecarlo chains, temperature set to 0.2, and sampling 10 million generations (standard deviation ≤ 0.1) with trees sampled every 1000 generations. The first 25% of the samples were discarded as burn-in, and stationarity was checked in Tracer v. 1.6 [67]. Trees were visualized and optimized in FigTree v. 1.4.4 [67] and edited in Adobe Illustrator vCS4 (Adobe Systems, Inc., San Jose, CA, USA).

## 3. Results

### Taxonomy

Agaricomycetes, Agaricales, Hymenogastraceae

*Gymnopilus guzmanii* R. Valenz., Baut.-Hern., and Raymundo sp. nov.

MycoBank: MB842046

**Figure 2:**
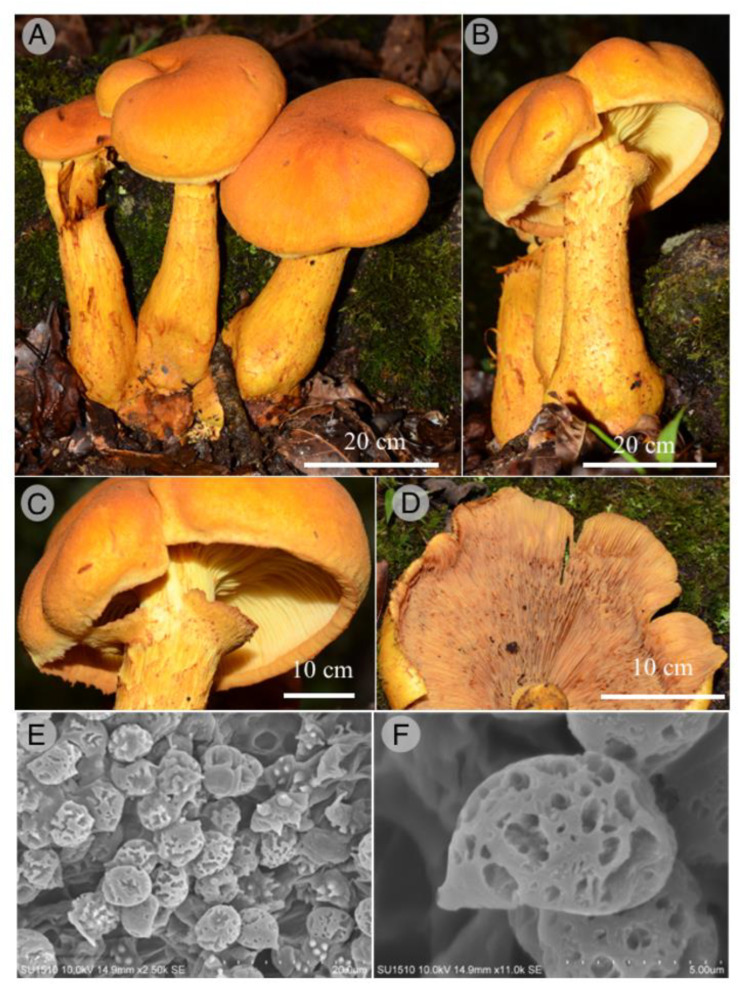
*Gymnopilus guzmanii*. (**A**,**B**): basidiomata; (**C**,**D**): detail of annulus and hymenophore; (**E**,**F**): SEM of basidiospores.

**Figure 3:**
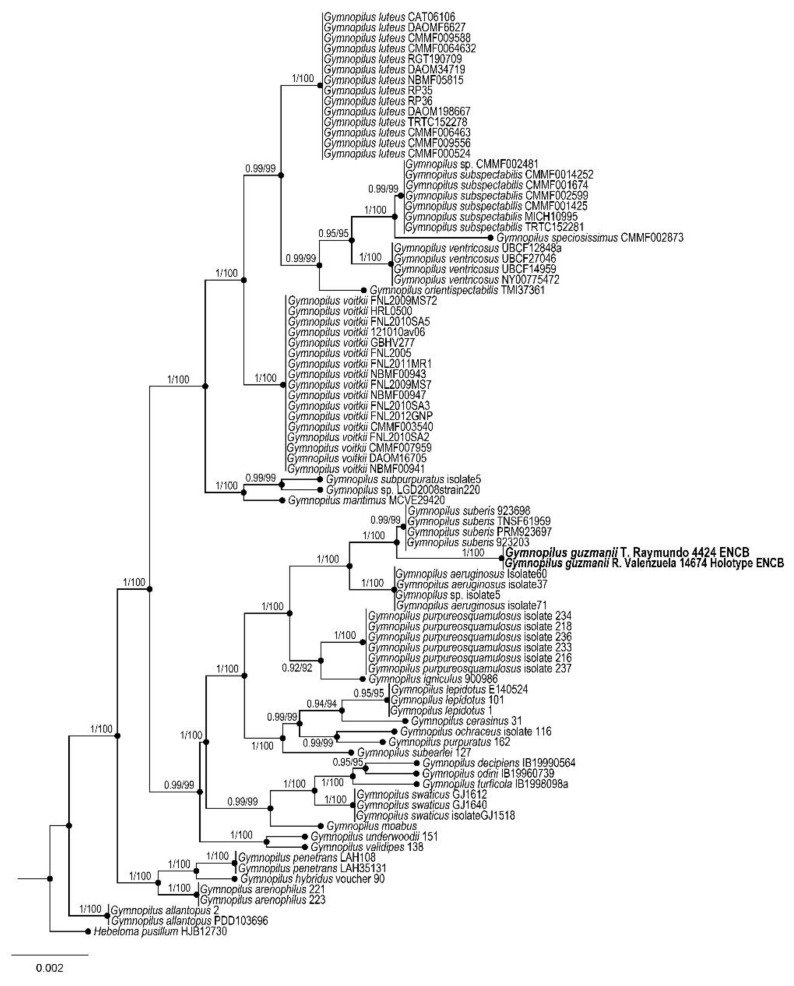
Bayesian inference phylogram of ITS sequences data. Posterior probability (**left** of slash) from Bayesian analysis and bootstrap support (**right** of slash). The new species *Gymnopilus guzmanii* is shown in bold. Boldface names represent samples sequenced for this study.

Figures: Figure 2 and Figure 3

Diagnosis: This species is different from other large annulate *Gymnopilus* species by its basidiomata growing in caespitose clusters on the ground of TMCF with fibrillose-squamulose, yellowish-orange to orange-red pileus and microscopically, basidiospores 7–9 × 5.5–7 µm, broadly ellipsoid, subreticulate to coarsely roughened with irregular large warts and ridges.

**Figure 4:**
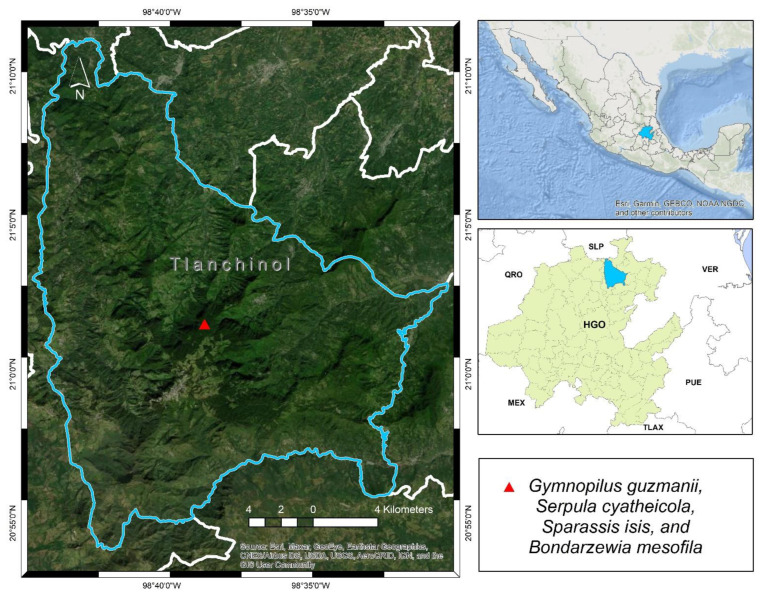
Distributional map of the species. Mexican states abbreviations are as follows: HGO, Hidalgo; PUE, Puebla; QRO, Querétaro; SLP, San Luis Potosí; TLAX, Tlaxcala, and VER, Veracruz.

Type: MÉXICO: Hidalgo state, Tlanchinol municipality, El Temazate, 21°01′40″ N, 98°38′33″ W, 1500 m, 18 July 2012, R. Valenzuela 14674 (ENCB, Holotype). Figure 4. 

Genbank: ITS: OW764567.

Etymology: This species is dedicated to Dr. Gastón Guzmán, a Mexican mycologist pioneer in studying macrofungi in México.

Pileus 50–200 mm diameter, convex when young, then plane-convex to depressed at the center in mature specimens, yellowish orange (4B7) to light orange (5A5) or reddish orange (7B7) when young; yellowish orange (4B7), orange (6B7) to reddish orange (7B7) to the margin; and brownish orange (7C8), orange red (8B7), red pastel (8A5) to brownish red (8C7), dark red (10C8) with KOH 5%, dry, fibrillose-squamulose, margin appendiculate in young specimens, eroded in some parts, recurved in mature specimens; lamellae adnate to subdecurrent, vivid yellow (3A8) or deep yellow (4A8) when young; yellowish brown (5E8) in mature specimens; close, broad, up to 20 mm, smooth edge, deep yellow (4A8) to orange yellow (4B8), releasing a yellow pigment in KOH on the slide; stipe 60–150 × 10–20 mm, clavate to subbulbous (20–40 mm broad at the base), yellowish orange (4B7), orange (6B7) to reddish orange (7B7) or brownish orange (7C8), solid, annulate, smooth at the apex, fibrillose to fibrillose-adpressed under the annulus, longitudinally striate; partial veil well developed, leaving a subapical membranous or fibrillar annulus in young specimens and fading in mature specimens, deep yellow (4A8) to yellowish orange (4B7); context up to 15 mm thick, fleshy, vivid yellow (3A8) to yellowish orange (4B7); basidiospores 7–9 × 5.5–7 µm, reddish yellow, yellowish brown to reddish brown in KOH 5%, broadly ellipsoid, subreticulate to coarsely roughened with large irregular warts and ridges (up to 1 µm high); basidia 25–29 × 7–9 µm, 4-spored, yellowish to yellowish brown in KOH 5%; sterigmas 3–6 µm height; pleurocystidia 25–35 × 7–9 µm, ventricose, reddish brown in KOH 5%, slightly thick walled; cheilocystidia 37–44 × 5–7 µm, capitate, yellow to yellowish brown in KOH 5%; hymenophoral trama subparallel, with hyaline, yellow to yellowish brown hyphae, 4–15 µm, clamp connections present; pileipellis a cutis with postrate hyphae, yellow to reddish brown in KOH 5%, 5–12 µm diameter, clamp connections present, squamules with prostrate to suberect hyphae, reddish brown in KOH; pileocystidia and caulocystidia not observed.

Habitat: Gregarious to caespitose in TMCF soil.

Taxonomical notes: *Gymnopilus guzmanii* is characterized by its large annulate basidiomes growing in caespitose clusters on the soil of the tropical montane cloud forest, with fibrillose-squamulose, yellowish orange to orange-red pileus, margin appendiculate, eroded and recurved. Microscopically, basidiospores differ with subreticulate to coarsely roughened ornamentation with irregular large warts and ridges. Morphological and molecular characters locate the species in the *G*. *junonius* (Fr.) P.D. Orton group [68]. Phylogenetically, *G*. *guzmanii* is well-supported based on ITS sequence data. It is close to *G*. *subspectabilis* Hesler and *G*. *ventricosus* (Earle) Hesler. The first species grows on hardwoods, while the second grows on the wood of conifers. Other characteristics that separate these species are the shape and ornamentation of spores in *G*. *subspectabilis*, ellipsoidal to amygdaliform, with acutely conical apices and conspicuous suprahylar depressions, moderately roughened with irregular warts and short ridges. In *G. ventricosus,* the spores are amygdaloid, with conical apices, finely to coarsely roughened with irregular warts and ridges.

Boletales, Serpulaceae 

*Serpula cyatheicola* Raymundo, de la Fuente, García-Jiménez, Martínez-González, and R. Valenz. sp. nov.

MycoBank: MB842039 

**Figure 5:**
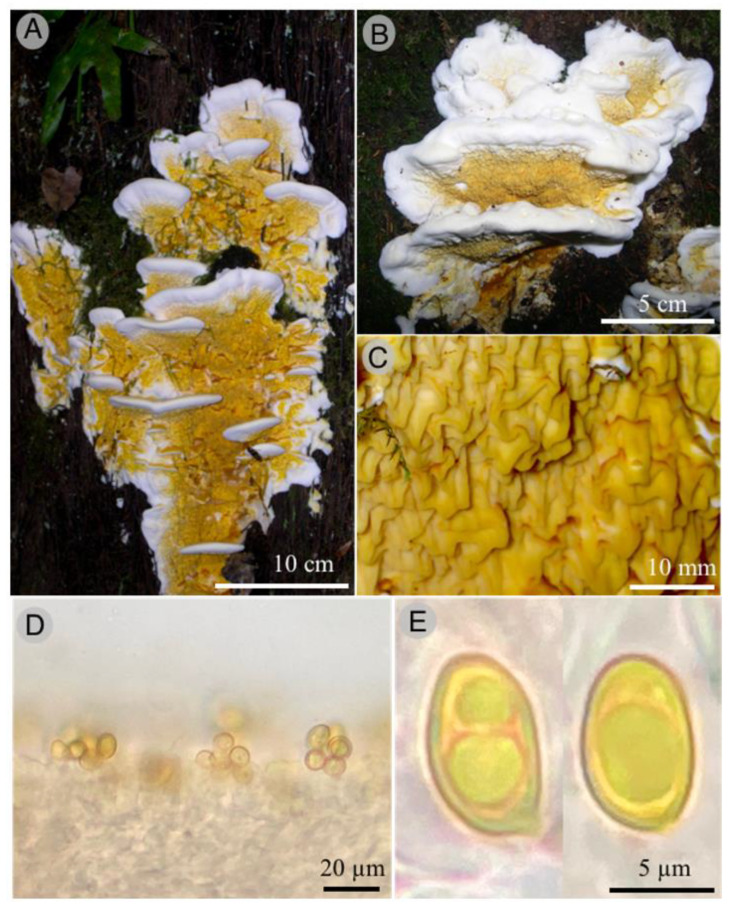
*Serpula cyatheicola*. (**A**,**B**): basidiomata; (**C**): detail of hymenophore; (**D**): hymenium; (**E**): optical microscope images of basidiospores.

**Figure 6:**
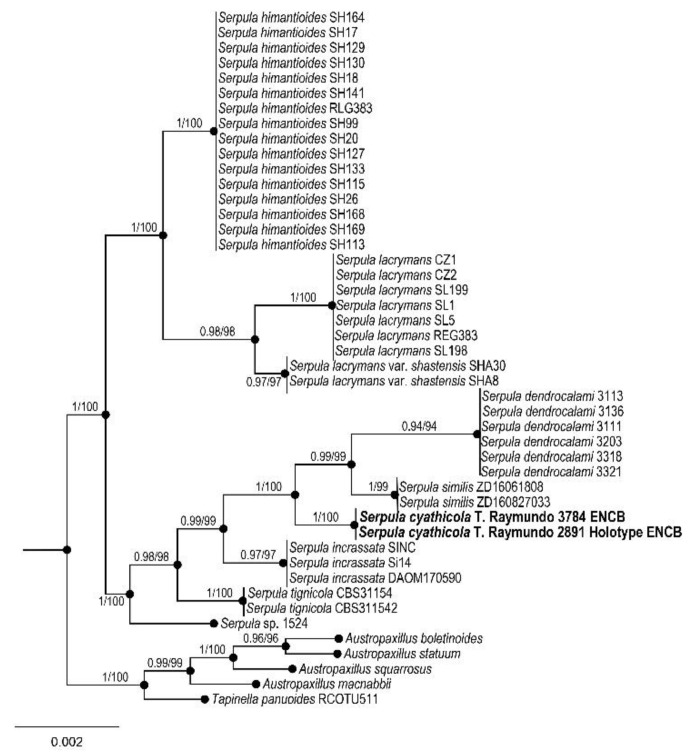
Bayesian inference phylogram of ITS sequences data. Posterior probability (**left** of slash) from Bayesian analysis and Bootstrap support (**right** of slash). The new species *Serpula cyatheicola* is shown in bold. Boldface names represent samples sequenced for this study.

Figures: Figure 5 and Figure 6

Diagnosis: *Serpula cyatheicola* is characterized by the ellipsoid basidiospores of 7.5–8 × 4.5–5 µm, clavate basidia up to 50 µm long, growing over *Cyathea mexicana* Schltdl. and Cham.

Type: MÉXICO: Hidalgo state, Tlanchinol municipality, El Temazate, 21°01′40″ N, 98°38′33″ W, 1500 m, 24 July 2009, T. Raymundo 2891 (ENCB Holotype). Figure 4.

GenBank: ITS: OW764589.

Etymology: The epithet *cyatheicola* was given because it grows on *Cyathea mexicana.*

Basidiomata annual, 50–100 × 30–70 × 10–12 mm, resupinate, dimidiate to effuse-reflexed, pileate to sessile, cartilaginous to spongy, cadmium (4A8), chromo (5A8), with white margin (4A1); pileus semicircular, with thick sterile margin; context spongy, white; hymenophore merulioid with folds of 400–420 µm thick, in fresh deep-yellow (4B8), rust-brown (6E8) when dry; hyphal system monomitic, with generative hyphae of 3–4 µm diameter, with clamp-connections; hymenophoral trama parallel, 800–1000 µm diameter, composed of hyaline interwoven hyphae; subhymenium 40–46 µm diameter; hymenium 80–88 µm diameter, with clavate basidia of 46–50 × 9–10 µm, hyaline, with short sterigmata of 3–4 µm, thin-walled; basidiospores 7.5–8 × 4.5–5 µm, ellipsoid, brown in KOH, some guttulate, smooth, thin-walled. 

Habitat: growing on *Cyathea mexicana* in the TMCF.

Additional specimens examined: MÉXICO: Hidalgo state, Tlanchinol municipality, Lontla, growing on *Cyathea mexicana*, 21°01′40″ N, 98°38′34″ W, 24 July 2009, T. Raymundo 3784 (ENCB); 27 June 2009, J. García-Jiménez 17975 (ITCV); 28 July 2014, J. García-Jiménez 19866 (ITCV).

Taxonomical notes: The order Boletales is a group of fungi within the Agaricomycetes typically characterized by the putrescent boletoid fruit body with poroid hymenia [50]. Nowadays, the order Boletales groups boletoid lineages and lamellate, sequestrate, and resupinate species [51,69]. Most of the typical boletoid species form ectomycorrhizal associations with Pinaceae, Fagaceae, Betulaceae, Dipterocarpaceae, and Polygonaceae [40], but some are saprobic and parasitic. The molecular evidence shows that the basal clades in the Boletales, such as the Coniophoraceae, Tapinellaceae, and Serpulaceae families, are composed of resupinate species [70,71,72]. The species of *Serpula* (Pers.) Gray are characterized by resupinate to rarely pileate fruit bodies with poroid or merulioid hymenia [58,73,74]. Microscopically, the genus is characterized by a monomitic hyphal system, ellipsoid to ovoid, and smooth, thick-walled, brownish basidiospores [58,73]. The mycorrhizal genus *Austropaxillus* Bresinsky and Jarosch and the sequestrate *Gymnopaxillus* E. Horak form the Serpulaceae family [58]. The species of this genus are distributed worldwide; about 17 species are known, but some species complexes may exist [58,59,75]. Furthermore, some species cause structural damage to wooden houses [59,60,75,76]. *Serpula cyatheicola* is characterized mainly by the ellipsoid basidiospores of 7.5–8 × 4.5–5 µm, clavate basidia up to 50 µm long, and living on *Cyathea*. It differs from *S*. *lacrymans* (Wulfen) J.Schröt. by the smaller basidia (up to 37 µm long), absence of rhizomorphs, and longer basidiospores (7–12 × 4–8 µm) [59,75]; this species was recorded previously from Mexico [61,77]. *Serpula dendrocalami* C.L. Zhao also have large pileus and merulioid pores but differ in the smaller basidiospore size (4.5–5.5 × 3.5–4 µm) and the habit on the roots of *Dendrocalamus* [58]. The basidiospore size of the new species resembles those from *Leucogyrophana hexagonoides* (Burt) Domański (=*S*. *hexagonoides* (Burt) W.B. Cooke) and *S*. *similis* (Berk. and Broome) Ginns. Nevertheless, the latter species grows on *Sequoia* sp. on sandy soils [73,78]. The length of the basidiospores of *Hydnomerulius pinastri* (Fr.) Jarosch and Besl (=*S*. *pinastri* (Fr.) W.B. Cooke) are similar to those from *S*. *cyatheicola*. However, the latter species grows on *Pinus*, *Abies*, and *Quercus* and has smaller basidia (18–20 × 4–5 µm) [73].

Polyporales, Sparassidaceae

*Sparassis isis* Raymundo and R. Valenz. sp. nov.

MycoBank: MB842052 

**Figure 7:**
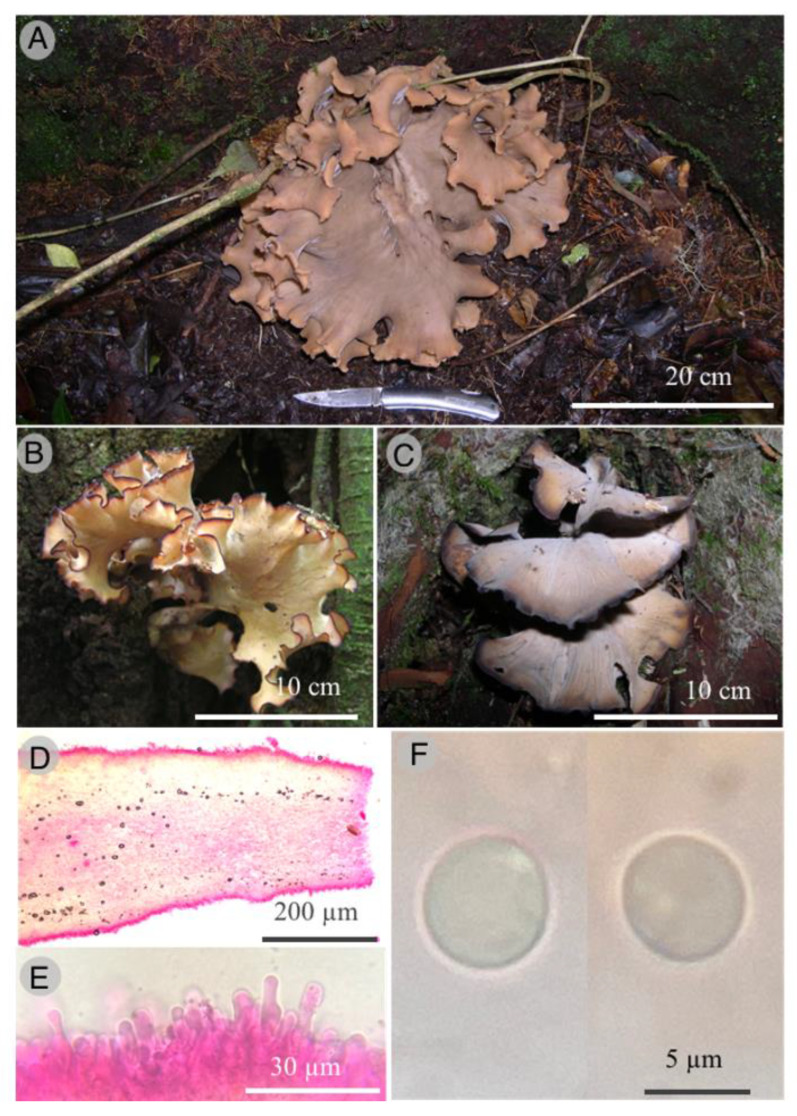
*Sparassis isis*. (**A**–**C**): basidiomata; (**D**): optical microscope images of the hymeniiferous trama; (**E**): optical microscope images of hymenium; (**F**): optical microscope images of basidiospores.

**Figure 8:**
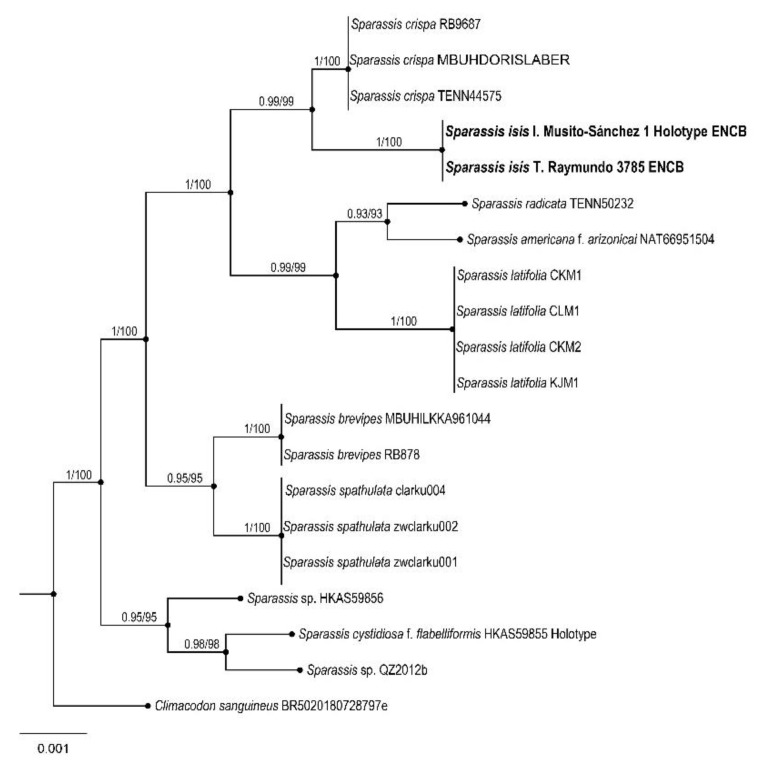
Bayesian inference phylogram ITS, LSU, *rpb2,* and *atp6* sequences data. Posterior probability (**left** of slash) from Bayesian analysis and bootstrap support (**right** of slash). The new species *Sparassis isis* is shown in bold. Boldface names represent samples sequenced in this study.

Figures: Figure 7 and Figure 8

Diagnosis: This species is different from other species by its long basidiomata (up to 400 mm diameter and 200 mm high), composed of one to four layers of loosely arranged very broad flabellae with very wavy, zonate, darkening with age margins, cystidia absent, but cystidioles present, and basidiospores 5–7 × 4–6 μm, globose to subglobose.

Type: MÉXICO: Hidalgo state, Tlanchinol municipality, El Temazate, 15 May 2008, 21°01′40″ N, 98°38′34″ W, Isis Musito-Sánchez *s.n*. (Holotype: ENCB). Figure 4.

GenBank: ITS: OW769404; LSU: OW769865; *rpb*2: OW769853; *atp*6: OW769812.

Etymology: This species is dedicated to Isis Musito Sánchez (*in memoriam*), who collected the specimen type.

Basidiomata (100-) 200–400 mm diameter, 100–200 mm high, annual, solitary, composed of one to four layers of loosely arranged flabellae arising from a poorly developed central core; flabellae (70-) 100–250 mm broad, mostly mainly extend from a common central mass, contorted, semicircular to flaveliform, upper surface glabrous, light brown (6D5), cinnamon brown (6D6) to grayish brown (6D3), towards the margin grayish orange (6B5) to brownish orange (6C5), becoming brownish gray (6D3) to grayish brown (6E4) with age or when dry; top margin entire, waxy, wavy, zonate, darkening with age, becoming brown, dark brown to black; hymenial surface pale yellow (4A3), light yellow (4A4) to grayish orange (6B5) or brownish orange (6C5); basidiospores 5–7 × 4–6 μm, globose to subglobose, hyaline, thin-walled, smooth, non-amyloid; basidia 20–30 × 5–6 μm, clavate, 4-spored, hyaline, usually with a basal clamp connection, sterigmata 4–7 μm long; cystidia absent, cystidioles 15–20 × 5–7 μm, sublageniform to clavate, hyaline; subhymenium composed of tightly packed, thin-walled, hyaline, ramose-inflated hyphal elements; trama of flabellae forming a monomitic hyphal system, composed of loosely interwoven hyphae embedded in a gelatinous or mucilaginous matrix, hyaline, inamyloid, thin to thick-walled, 4–6 μm diameter, commonly clamped; hyphae gloeoplerous scattered throughout, refractive, flexuous, aseptate, thin-walled, 6–12 μm diameter, most abundant towards the base of the basidiomata.

Habitat: This species grows at the base of living *Quercus* trees, attached to underground roots in the TMCF.

Additional specimens examined: Paratypes. MÉXICO: Hidalgo state, municipality of Tlanchinol, El Temazate, 7 June 2009, 21°01′40″ N, 98°38′34″ W, R. Valenzuela 13895 (ENCB); 22 August 2011, T. Raymundo 3785 (ENCB).

Taxonomical notes: *Sparassis isis* is characterized by its basidiomata composed of a few layers of loosely arranged flabellae, very wavy and darkening margins, trama of flabellae composed of hyphae embedded in a gelatinous or mucilaginous matrix, cystidioles present, and for its size and shape of basidiospores. This species is separated from other *Sparassis* by morphological, ecological, and molecular characters and phylogenetically is well supported based on ITS, LSU, *rpb2*, and *atp6* sequences [63,64,65,79,80,81]. *Sparassis isis* belongs to the North American clade with *S*. *americana* R. H. Petersen, *S*. *radicata* Weir, and an unidentified species. *S. americana* differs by its upper basidiome coarsely and irregularly branched to produce expanded petaloid crisped flabellae, top margins drying cartilaginous when fresh appearing waxy, retaining this appearance when dried; basidiospores 4.5–6 (7.0) × (3.0) 3.5–4.0 (4.5) μm, broadly ellipsoid and flattened axially and associated with *Pinus* sp., perhaps as root parasite [59,66]. *S. radicata* is separated by its subterranean pseudosclerotial stipe, an above-ground fertile structure composed of complex, often anastomosed lacunose branches, with ultimate flabellae thin, parchment-like in well-dried specimens, curled or crisped, basidiospores 5–6.5 × 3.5–4 (5) μm, broadly ellipsoid to ellipsoid and flattened axially. It is associated with the roots of conifers in North American temperate forests [59].

Russulales, Bondarzewiaceae

*Bondarzewia mesofila* R. Valenz., Baut.-Hern., and Raymundo sp. nov. 

MycoBank: 842,140 

**Figure 9:**
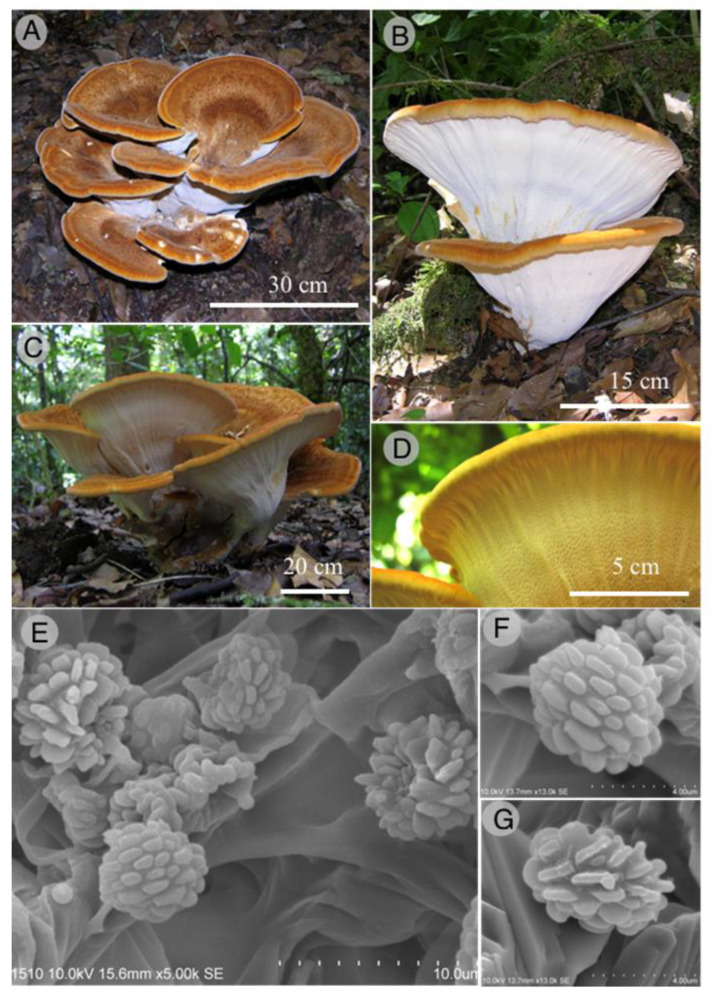
*Bondarzewia mesofila*. (**A**): basidiomata; (**B**,**C**): hymenophore; (**D**): detail of hymenophore; (**E**–**G**): SEM of basidiospores.

**Figure 10:**
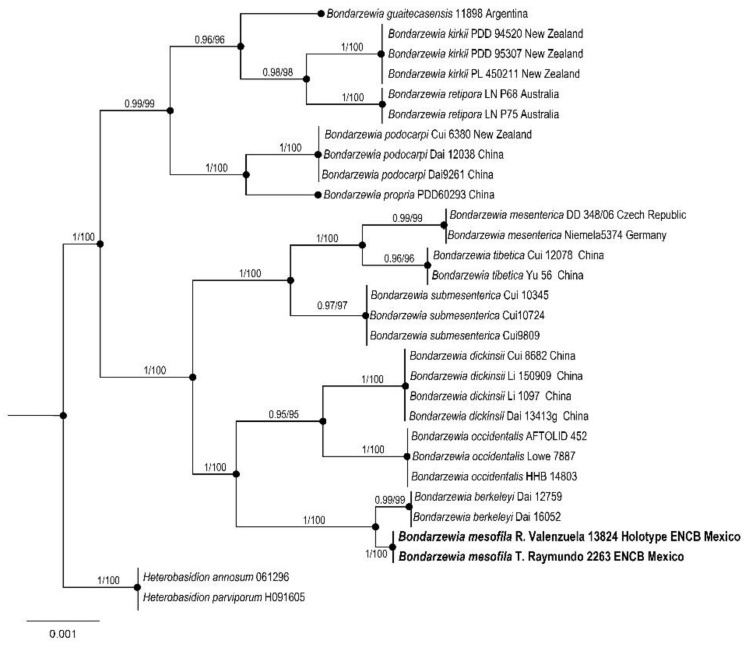
Bayesian inference phylogram ITS, LSU, SSU, and *tef1* sequences data. Posterior probability (**left** of slash) from Bayesian analysis and bootstrap support (**right** of slash). The new species *Bondarzewia mesofila* is shown in bold. Boldface names represent samples sequenced for this study.

Figures: Figure 9 and Figure 10

Diagnosis: It differs from other *Bondarzewia* species in having pileus in several tones, e.g., orange, yellow ochre, brownish yellow, light brown, yellowish brown or rust brown, tomentose to hirsute and towards the margin tomentose adpressed, margin white, yellow to orange, most extended basidiospore ridges, up to 2 μm; basidiomata grows on the soil.

Type: MÉXICO, Hidalgo state, Tlanchinol municipality, El Temazate, 6 June 2009, 21°01′40″ N, 98°38′34″ W, R. Valenzuela 13824 (Holotype: ENCB). Figure 4.

GenBank: ITS: OW768521; LSU: OW768975; SSU: OW768127; *tef1:* OW769940

Etymology: The epithet of the species refers to the vegetation type where it grows (“bosque mesófilo de montaña”). 

Basidiomata 200–350 mm wide, 200–250 mm high, annual, pileate stipitate, imbricate or slightly imbricate in some specimens, fleshy when fresh, becoming corky to hard in dry specimes, odor pleasant and taste similar to walnut or almonds; pileus (100-) 150–250 mm long, 100–200 mm wide and up to 15 mm thick, semicircular to flabelliform, several tones of orange (5AB8), yellow-ochre (5C7), brownish yellow (5C8), light brown (5D8), yellowish brown (5EF8), rust brown (6E8), azonate to obscurely zonate in the middle, zonate to the margin, glabrous to velvety in young specimens or in the center of mature specimens, tomentose to hirsute in the middle, and towards the margin tomentose adpressed; margin obtuse, sterile, velvety, white (4A1), yellow (4A6) to orange (5AB8); hymenophore poroid, white (4A1) to cream (4A3) in young specimens, becoming light yellow (4A5), stained with yellowish brown (5D8) when mature, pores 1–2 per mm, angular to elongate (up to 2 mm long) or irregular in shape, tubes shallow to the margin, up to 2 mm deep, concolorous with the pores, dissepiments thin, entire to lacerate; stipe up to 80 mm and 40 mm wide, white (4A1) to yellow (4A6) when young specimens, yellow ochre (5C7), light brown (5D8) to yellowish brown (5EF8) in mature specimens, rust brown (6E8), velvety; context up to 12 mm thick, white (4A1) to yellow ochre (5C7), fleshy in fresh, corky when dry; hyphal system monomitic in trama, dimitic in context; generative hyphae simple septate, thin-walled, skeletal hyphae unbranched, thick walled,, not septated, 5–7 μm diameter; contextual generative hyphae hyaline, thin-walled, simple septate, moderately branched, 5–7 μm diameter; contextual skeletal hyphae hyaline, thick-walled, interwoven, 6–7 μm diameter; trama generative hyphae interwoven, hyaline, thin-walled, simple septate, branched, 2–5 μm diameter; cystidia absent; basidia 17–20 × 7–9 μm, clavate, hyaline, with a simple basal septum, 4-spored; basidiospores 5.5–7 × (4) 4.5–6 μm, subglobose, rarely ellipsoid, hyaline, amyloid, thick-walled, with obvious ridges, ridges of spores blunt, up to 1 μm high and up to 2 μm long.

Habitat: *Bondarzewia mesofila* grows solitary on TMCF soil.

Additional specimens examined: MÉXICO: Hidalgo state, Tlanchinol municipality, El Temazate, 21°01′40″ N, 98°38′34″ W, 6 June 2009, T. Raymundo *2263* (Paratype: ENCB).

Taxonomical notes: This species is characterized by the color of basidiomata with several tones in the pileus (orange, yellow ochre, brownish yellow, light brown, yellowish brown, rust brown), margin (white, yellow to orange), and stipe (white, yellow, yellow ochre, light brown to yellowish brown), the texture of pileus (velvety, tomentose, hirsute and tomentose adpressed) and basidiospores ornamentation with ridges blunt, up to 1 μm high and up to 2 μm long. *Bondarzewia mesofila* can be separated by morphological, ecological, and molecular characteristics [67,82]. Phylogenetically is well supported based on ITS, LSU, SSU, and *tef*1 sequences. This species is close to *B*. *berkeleyi* (Fr.) Bondartsev and Singer, *B. dickinsii* (Berk.) Jia J. Chen, B.K. Cui, and Y.C. Dai, and to *B*. *occidentalis* Jia J. Chen, B.K. Cui, and Y.C. Dai, all of them belonging to a sister clade. *Bondarzewia berkeleyi* is separated by growing on the wood of several species of Fagaceae, basidiomata developing underground sclerotia, and larger basidiospores 7–9 × 6–8 μm [82,83]. Basidiomata of *Bondarzewia dickinsii* grow on fallen trunks of *Quercus* and roots of *Castanea*; the pileus color is white to brownish, basidiospores with sharp ridges, up to 2 μm long [82]. *Bondarzewia occidentalis* grows on gymnosperm wood, with a pileus surface characteristically from yellowish brown to orange-brown, concentrically zonate and glabrous, basidiospores with ridges up to 1 μm long [83].

## 4. Conclusions

Based on this study and other research performed by us and other colleagues, the Mexican TMCF is the most diverse ecosystem for fungi. Databases from different organisms, e.g., plants [84] and birds [85], are available for this ecosystem but not for fungi. Unfortunately, there are no extensive studies of fungi because of the lack of specialists, so their representation in the herbaria is poor. So far, 1407 species of Macromycetes have been registered for Mexico, of which 220 species have been found in this ecosystem. In addition, this study described four new species found in this ecosystem. Similar worldwide studies at the same latitude, e.g., in Yunnan, China [86,87], an area also considered as a biodiversity hotspot [88], recently recorded 314 macrofungi. Based on this, the Mexican TMCF could harbor more than 100 fungi species than those registered in this study.

The characterization of fungal diversity in TMCF is relevant for forest conservation. Fungi provide different environmental services and are sources of bioactive secondary metabolites [35]. Altogether, 1106 species of Agaricomycetes are registered from the TMCF [35]. This study contributes with four new species. 

Mexico represents one of the world’s most diverse areas for fungi diversity, so it is essential to record and describe the fungal species of this type of vegetation. TMCFs are the most threatened terrestrial ecosystems at the national level and are classified as “habitat in danger of extinction”. In addition, a meta-analysis recently revealed that Mexico is a hotspot for oak species and their ectomycorrhizal mycobionts [89]. These last authors considered the Mexican oak forest essential for maintaining biodiversity due to its high richness and endemism of fungi, mainly those associated with Fagaceae.

The loss of the TMCF is due to its transformation into grazing land for livestock and agriculture, mainly for avocado and citric. The fungal wealth is strongly affected by the loss of this ecosystem. The effects of global warming have not yet been evaluated in the case of fungi.

## Figures and Tables

**Table 1:** Primers used in the amplification and sequencing of the DNA fragments.

Loci/Segment	Primer	Sequence 5′-3′	T (°C)	Reference
ITS	ITS5	GGAAGTAAAAGTCGTAACAAGG	58	[48]
	ITS4	TCCTCCGCTTATTGATATGC	58	[48]
nLSU	LROR	ACCCGCTGAACTTAAGC	48	[48]
	LR3	GGTCCGTGTTTCAAGAC	48	[48]
*rpb2*	bRPB2-6R2	GARTGYCCDGGDCAYTTYGG	52	[49]
	bRPB2-7R2	CCNGCDATNTCRTTRTCCATRTA	52	[49]
*tef1*	EF1-B-F1	ATYGCTTTAGAAAGTTYMTTTGC	53	[50]
	EF1-B-R	GGDATRAARWAWGARAARAARTG	53	[50]
*atp6*	atp6-5	ATYGCTTTAGAAAGTTYMTTTGC	56	[51]
	atp6-6	GGDATRAARWAWGARAARAARTG	55	[51]
SSU	MS1	CAGCAGTCAAGAATATTAGTCAATG	63	[48]
	MS2	GCGGATTATCGAATTAAATAAC	*53*	[48]

**Table 2:** GenbBank accessions corresponding to the *Gymnopilus* sequences used in the phylogenetic analyses. In bold, the accessions of the new species. Species in bold correspond to the new accessions.

Species Name	Isolate/Voucher/Strain	GenBank Accessions
		ITS
*Gymnopilus aeruginosus* (Peck) Singer	Isolate 60	AY280975
	Isolate 71	AT254102
	Isolate 37	AY280974
*Gymnopilus cerasinus* (Sacc.) Guzm.-Dáv., G.M. Muell., J. Cifuentes, A.N. Mill., and Santerre	31	GH412552
*Gymnopilus igniculus* Deneyer, P.-A. Moreau and Wuilb.	900986	HY142500
***Gymnopilus guzmanii***	**T. Raymundo 4424**	**OQ749940**
	**R. Valenzuela 14674**	**OQ749941**
*Gymnopilus lepidotus* Hesler	E140524	YU415875
	101	UI475222
	1	HJ410256
*Gymnopilus luteus* (Peck.) Hesler	CAT06106	OP985111
	DAOMF6627	OM672062
	CMMF009588	MN477932
	CMMF0064632	MN477919
	RGT190709	MN477917
	DAOM34719	MN477293
	NBMF05815	MN453485
	RP35	MN453484
	RP36	MN453483
	DAOOM198668	MN718842
	TRTC152278	MN206894
	CMMF006463	MN206893
	CMMF009556	MN206892
	CMMF000524	MN206891
*Gymnopilus maritimus* Contu, Guzm.-Dáv., A. Ortega, and Vizzini	MCVE29420	JK210152
*Gymnopilus ochraceus* Høil.	Isolate 116	HJ125488
*Gymnopilus orientispectabilis* Nagas, Malloch, and Thorn	TM137361	YU125412
*Gymnopilus purpuratus* (Cooke and Massee) Singer	162	HJ140258
*Gymnopilus purpureosquamulosus* Høil.	Isolate 234	HJ521410
	Isolate 218	GH150236
	Isolate 236	GF852014
	Isolate 233	YG521400
	Isolate 216	GF251478
	Isolate 237	GB512014
*Gymnopilus speciosissimus* Y. Lamoureux, Malloch, and Thorn	CMMF002873	TG512152
*Gymnopilus suberis* (Maire) Singer	923698	GF250124
	TNSF61959	FG214585
	PRM923697	FF510535
	9232063	FV475210
*Gymnopilus subpurpuratus* Guzm.-Dáv. and Guzmán	Isolate 5	FG152024
*Gymnopilus subspectabilis* Hesler	CMMF001425	HB415204
	CMMF001674	BH125478
	CMMF002599	GH521402
	CMMF001425	HG251200
	MICH10995	BG520526
	TRTC152281	JH250145
*Gymnopilus ventricosus* (Earle) Hesler	UBCF12848a	GH250210
	UBCF27046	TY820152
	UBCF14959	HG521520
	NY00775472	GB520145
*Gymnopilus voitkii* Malloch and Thorn	FNL200MS72	HJ452145
	HRL0500	HN452104
	FNL2010SA5	UY541203
	121010av06	HN204789
	GBHV277	YH520384
	FNL2005	TG570134
	FNL2011MR1	NH521452
	NBMF00943	HJ251024
	FNL2009MS7	BN250145
	NBMF00947	GF204752
	FNL2010SA3	HJ520214
	FNL2012GNP	FV520145
	CMMF003540	GH102547
	FNL2010SA2	TY520147
	CMMF007959	VF520147
	DAOM16705	VB254054
	NBMF00941	VT012585

**Table 3:** GenbBank accession numbers corresponding to the *Serpula* sequences used in the phylogenetic analyses. In bold, the accessions of the new species.

Species Name	Isolate/Voucher/Strain	GenBank Accessions
		ITS
***Serpula cyatheicola***	**T. Raymundo 3784 ENCB**	**OQ749942**
	**T. Raymundo 2891 ENCB**	**OQ749943**
*Serpula dendrocalami* C.L. Zhao	3113	MK863408
	3136	MK863407
	3111	MK863406
	3203	MK863405
	3318	MK863404
	3321	MK863403
*Serpula himantioides* (Fr.) P. Karst.	SH164	LC710851
	SH17	LC710850
	SH129	LC710849
	SH130	LC710848
	SH18	LC710847
	SH141	LC710846
	RLG 383	LC710845
	SH99	LC710844
	SH20	LC710843
	SH127	LC710842
	SH133	LC710841
	SH115	LC637898
	SH26	LC637897
	SH168	LC637896
	SH169	LC637895
	SH113	LC637894
*Serpula incrassata* (Berk. and M.A. Curtis) Donk	SINC	HM135648
	Si14	HM135647
	DAOM 170590	GU187541
*Serpula lacrymans* (Wulfen) J. Schröt.	CZ1	GU066830
	CZ2	MW491273
	SL199	HB158420
	SL1	BV120158
	SL5	HV157863
	REG 383	HG547896
	SL 198	FG541256
*Serpula lacrymans* var. *shastensis* (Harmsen) Ginns and M.N.L. Lefebvre	SHA30	HD587452
	SHA8	TG558412
*Serpula similis* (Berk. and Broome) Ginns	ZD 16061808	MN523308
	ZD160827033	MN523307
*Serpula tignicola* (Harmsen) M.P. Christ.	CBS 31154	GU187543
	CBS 311542	HJ452102
*Serpula* sp.	1524	HN458520

**Table 4:** GenbBank accessions corresponding to the *Sparassis* sequences used in the phylogenetic analyses. In bold, the accessions of the new species.

Species Name	Isolate/Voucher/Strain	GenBank Accessions			
		ITS	nLSU	*rpb2*	*atp6*
*Sparassis americana* f. *arizonica* R.H. Petersen	NAT66951504	MW403771	MF693910	-----	-----
*Sparassis brevipes* Krombh.	RB878	MH861680	MH873392	AG452102	-----
	MBUHILKKA961044	KP100510	KP100511	AT412035	-----
*Sparassis crispa* (Wulfen) Fr.	RB9687	KJ754521	KJ850214	YD452110	-----
	MBUHDORISLABER	HJ541225	KL541252	-----	-----
	TENN44575	JK254102	KL458520	-----	AY452102
*Sparassis cystidiosa* f. *flabelliformis* Q. Zhao, Zhu L. Yang, and Y.C. Dai	HKAS59855	KY428924	-----	-----	-----
***Sparassis isis***	**I Musito-Sánchez 1**	**OQ749944**	**OQ747755**	**OX5210I7**	**OX52102I**
	**T. Raymundo 3785**	**OQ749945**	**OQ747756**	**OX5210I8**	**OX52I044**
*Sparassis latifolia* Y.C. Dai and Zheng Wang	CKM1	JK521452	HJ874520	AD478520	-----
	CLM1	LK521477	KJ514268	HG210548	-----
	CKM2	JI525455	IK870256	-----	JN452012
	KJM1	KL478601	KL472500	-----	HF420156
*Sparassis radicata* Weir	TENN50232	LK452102	KI210045	-----	JH021585
*Sparassis spathulata* (Schwein.) Fr.	Clarku004	LO478521	IO541256	HF478520	NB023575
	Zwclarku002	KJ457424	PO582114	GH021452	JV521452
	Zwclarku001	IK547888	OL452101	-----	-----
*Sparassis* sp.	QZ2012B	JI471250	OP852014	-----	-----
*Sparassis* sp.	HKAS59856	OK541200	KL457520	-----	-----

**Table 5:** GenbBank accessions corresponding to the *Bondarzewia* sequences used in the phylogenetic analyses. In bold, the accessions of the new species.

Species Name	Isolate/Voucher/Strain	GenBank Accessions			
		ITS	nLSU	*tef1*	SSU
*Bondarzewia berkeleyi* (Fr.) Bondartsev and Singer	Dai 12759	KJ583202	KJ583216	KX066138	KX066169
	Dai 16052	KX263720	KX263	-----	-----
*Bondarzewia dickinsii* (Berk.) Jia J. Chen, B.K. Cui, and Y.C. Dai	Cui 8682	KJ583209	KJ583223	KX066136	KX066167
	Dai 13413	KJ583210	KJ583224	KX066137	KX066168
	Li 150909/19	KX263721	KX263723	-----	-----
	Li 1097	FJ644288	-----	-----	-----
*Bondarzewia guaitecasensis* (Henn.) J.E. Wright	Rajchenberg 11898	FJ644287	-----	-----	KX066175
*Bondarzewia kirkii* J.A. Cooper, Jia J. Chen, and B.K. Cui	PDD 94520	KJ583215	KJ583229	KX252748	KX066180
	JAC 10839	KJ734674	KM067469	KX252747	KX066179
	PL 450211	KJ583214	KJ583228	KX252746	KX066178
*Bondarzewia mesenterica* (Schaeff.) Kreisel	DD 348/06	KM243328	KM243331	KX066147	KX066182
	Niemelä	KM067468	KM067470	KX066146	KX066181
***Bondarzewia mesofila***	**R. Valenzuela 13824**	**OQ749938**	**OQ747753**	**OQ5210I4**	**OQ749946**
	**T. Raymundo 2263**	**OQ749939**	**OQ747754**	**OQ52I027**	**OQ749947**
*Bondarzewia occidentalis* Jia J. Chen, B.K. Cui, and Y.C. Dai	Lowe 7887	KM243330	KM243333	KX066143	KX066177
	HHB 14803	KM243329	KM243332	KX066142	KX066176
	AFTOL-ID 452	DQ200923	DQ234539	DQ059044	-----
*Bondarzewia podocarpi* Y.C. Dai and B.K. Cui	Cui 6380	KJ583206	KJ583220	KX252745	KX066174
	Dai 9261	KJ583207	KJ583221	KX252743	KX066172
	Dai 12038	KJ583208	KJ583222	KX252744	KX066173
*Bondarzewia propria* (Lloyd) J.A. Cooper	PDD 60293	KJ583213	KJ583227	-----	-----
*Bondarzewia retipora* (Cooke) M.D. Barrett	LNP 68	KJ747633	KJ747630	-----	KX066144
	LNP 75	KJ747632	KJ747629	-----	KX066145
*Bondarzewia submesenterica* Jia J. Chen, B.K. Cui, and Y.C. Dai	Cui 9809	KJ583203	KJ583217	KX066141	KX066171
	Cui 10345	KJ583204	KJ583218	KX066140	KX066170
	Cui 10724	KJ583205	KJ583219	KX066139	-----
*Bondarzewia tibetica* B.K. Cui, J. Song, and Jia J. Chen	Cui 12078	KT693020	KT693204	KX066149	KX066184
	Yu 56	KT693203	KT693205	KX066148	KX066183
*Heterobasidion annosum* (Fr.) Bref.	06129/6	KJ583211	KJ583225	KX252741	KJ651577
*Heterobasidion parviporum* Niemelä and Korhonen	H 091605	KJ651503	KJ651561	KX252742	KJ651622

## Data Availability

Not applicable.
